# Simultaneous In Vivo Electrophysiology, Two-Photon Imaging, and Optogenetics for Probing Neurovascular Coupling

**DOI:** 10.3390/mps9030068

**Published:** 2026-04-25

**Authors:** Dalchand Ahirwar, Kun Xie, Philip O’Herron

**Affiliations:** Department of Physiology, Augusta University, Augusta, GA 30912, USA; dahirwar@augusta.edu (D.A.);

**Keywords:** electrophysiology, two-photon imaging, multimodal imaging, neurovascular coupling, functional hyperemia, neural dynamics, local field potential

## Abstract

Neuronal activity and cerebral blood flow are tightly coupled to support the high metabolic demands of the brain. Disruption of neurovascular coupling is a defining feature of many neurodegenerative disorders such as Alzheimer’s disease, stroke, small vessel disease, Parkinson’s disease, and aging. Progress in understanding the mechanisms underlying neurovascular coupling requires experimental approaches that can simultaneously measure neuronal activity and vascular dynamics with high spatial and temporal resolution, while also enabling targeted perturbations of the system. Here, we present a methodological framework that combines chronic electrophysiological recordings with two-photon imaging of cerebral blood flow and optogenetic manipulation of the vasculature in vivo. Using a chronically implanted flexible electrode array, we obtain measurements of the single- and multi-unit spiking activity, as well as local field potentials. Concurrently, two-photon microscopy enables high-resolution measurements of vessel diameter and blood flow within individual vascular segments. In addition, optogenetic control of vascular smooth muscle cells allows for rapid and reversible manipulation of the vessel diameter through the same cranial window while simultaneously recording the neural and vascular activity. We provide detailed protocols for surgical implantation, data acquisition, and analysis, and discuss experimental considerations and limitations. This combined platform offers a powerful tool for mechanistic studies of neurovascular coupling and its dysfunction in disease models.

## 1. Introduction

Neurons are highly metabolically demanding cells that rely on a continuous supply of oxygen and nutrients delivered by the cerebral vasculature [1,2,3]. Beyond nutrient delivery, blood vessels support brain function by regulating molecular transport across the blood–brain barrier, maintaining stable perfusion across fluctuations in systemic blood pressure (autoregulation), and clearing metabolic waste products [4,5,6,7,8]. Disruption of any of these processes contributes to tissue dysfunction and is a hallmark of numerous neurological diseases, including Alzheimer’s disease, vascular dementia, small-vessel pathologies, and ischemic injury [9,10,11,12,13,14]. Understanding how neuronal activity interacts with vascular dynamics, therefore, requires methods that are capable of monitoring both neuronal and hemodynamic signals simultaneously [15].

Chronic cranial windows provide a robust platform for achieving this, offering stable and repeatable optical access to the cortex for combined neural and vascular imaging with high spatial resolution [16,17]. Through these windows, vascular dynamics can be monitored by using wide-field approaches such as laser speckle contrast imaging, which enables rapid, mesoscale mapping of blood flow across the cortical surface [18]. At a higher resolution, two-photon microscopy allows for detailed measurements of the vessel diameter and red blood cell velocity within individual vascular segments [19,20]. Two-photon microscopy is also widely used to monitor neural activity—most commonly with genetically encoded calcium indicators—which enables population and single-cell measurements deep in the cortex [21]. However, calcium indicators provide only an indirect measure of spiking and lack the temporal precision of electrical recordings. In addition, network-level electrical signals—such as low-frequency oscillations measured with the local field potential (LFP)—cannot be readily inferred from calcium imaging. Genetically encoded voltage indicators offer a more direct readout of membrane potential, but require specialized high-speed imaging systems and remain limited by rapid photobleaching [22]. More broadly, all fluorescence-based measurements of neural activity are susceptible to artifacts such as photobleaching, sensor saturation, variable expression levels, and optical interference from the vasculature. Consequently, electrophysiological recordings remain the gold standard for precise neuronal activity measurements [23,24].

In addition to monitoring the activity of neurons and blood vessels, the ability to experimentally manipulate this activity is a powerful means to better understand neurovascular interactions. We recently developed an approach to optogenetically manipulate vascular smooth muscle cells, enabling rapid, reversible control of the vessel diameter using light delivered through the cranial window [25]. Such targeted perturbations allow for direct testing of how vascular changes influence neural activity and circuit function, and when combined with two-photon imaging and electrophysiology, comprises a powerful toolset for studying neurovascular interactions. While the combination of electrical and optical approaches through transparent cranial windows is not novel [26,27,28], many applications involve surface-only electrode contacts and have not been made widely commercially available. Furthermore, we are not aware of any protocol that combines recordings from electrodes inserted into the parenchyma, optogenetics, and two-photon imaging at depth.

Here, we present a methodological framework that integrates electrophysiological recordings of neural activity with two-photon imaging of blood flow and optogenetic manipulation of the cerebral vasculature. Our detailed step-by-step protocol using commercially available equipment will allow for easy replication. We use chronically implanted flexible electrodes, as originally developed by Refs. [29,30,31], to measure single- and multi-unit spiking activity and local-field potentials throughout the layers of the cortex. This combination enables the simultaneous measurement and perturbation of neurovascular dynamics with high spatial and temporal precision, providing a platform for directly testing the mechanisms linking neural activity, vascular responses, and cortical circuit function. We provide detailed descriptions for all steps, including surgery (installing the cranial window, electrode implantation), data collection (recording neuronal and vascular activity, optogenetically manipulating vessels), and data analysis. The variations and limitations of the approach are discussed.

## 2. Experimental Design

Our protocol provides detailed steps for the major aspects of the experiments, including the cranial window surgery, the electrode placement, and the imaging/recording experiment. We include detailed lists of the surgical equipment (Table 1), all liquid solutions used (Table 2), and the components of the electrophysiology (Table 3) and two-photon imaging (Table 4) systems. All handling, housing, and surgical procedures were approved by Augusta University Animal Care and Use Committee (AUP#2018-0982). Many aspects have been described previously [25]. During anesthetized imaging, mice breathed isoflurane (0.7–0.8%) in 0.5 L/min air and were injected with ~30 µL chlorprothixene [25]. Functional hyperemia was evoked through visual stimulation, leading to increased neuronal activity and blood vessel diameter in all animals [19,21,25,32]. For optogenetic vasoconstriction, we crossed a PDGFRβ-Cre mouse line with a Cre-dependent ReaChR line, as described in detail in (O’Herron et al. 2022) [25]. A summary of the overall timing of each experimental section can be found in Supplementary Figure S3.

## 3. Procedure

### 3.1. Pre-Surgical Steps

**Figure 1:**
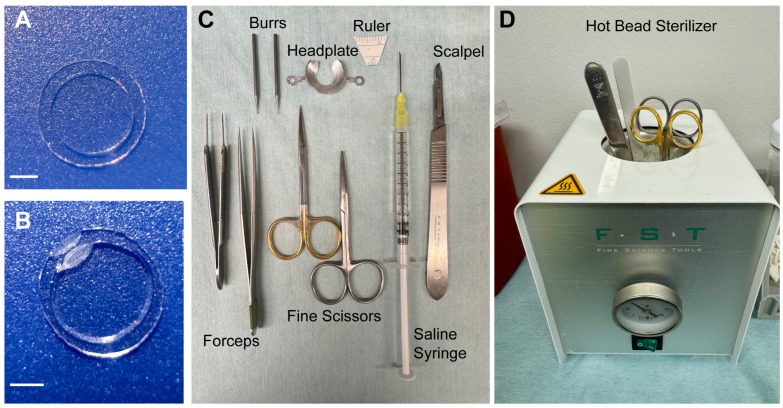
(**A**). The 3 mm cover glass glued to 4 mm cover glass. Scale bar, 1 mm. (**B**). Double-layered cover glass after beveling along the edge. (**C**). List of tools used during surgery. (**D**). The sterilization of tools.

1. The 3D CAD tool file for the headplate we use (Supplementary Figure S1) is provided in Supplementary Material S2. After completing the headplate design in the 3D software, the file is sent to the supplier for machining with aluminum.
2. Prepare anesthesia stock solution: 0.15 cc Ketamine (100 mg/mL), 0.25 mL Dexmedetomidine (0.5 mg/mL), and 0.6 mL saline.
3. Prepare anesthesia antidote stock solution: 0.25 mL Antisedan (2.5 mg/kg, 1.25 mg/mL) and 0.75 mL saline.
4. Make a double-layered cover glass by gluing a 3 mm cover glass to a 4 mm cover glass, using UV-cured glue (Figure 1A). Then, bevel a spot with a grindstone drill bit to create a gap for the electrode to lie in between the skull and the cover glass (Figure 1B).
5. Cut a gap between the arms of the headplate to create an opening for electrode placement (Figure 1C).

6. Prepare the surgical tools: two fine scissors, one suture tying forceps, one Dumont #3 forceps, one Dumont #5CO forceps, scalpel handle #3, #3 scalpel blade, two 19007-05 burrs, and animal headplate (Figure 1C). Autoclave or place them in the hot bead sterilizer for sterile surgery (Figure 1D).
7.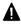 **CRITICAL STEP:** Autoclaving the 19007-05 burrs for the micro drill leads to rust accumulation. Use of the hot bead sterilizer for these is recommended.
8. Weigh the mouse and administer the Dexamethasone IM 0.03 mL/30 g and wait for 2–4 h before beginning surgery.
9. Turn on the heating pad and vacuum tube.
10.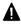 **CRITICAL STEP:** Ensure the vacuum pressure is not so high as to cause damage to the tissue.

### 3.2. Preparing the Craniotomy Window (10–20 Min)

**Figure 2:**
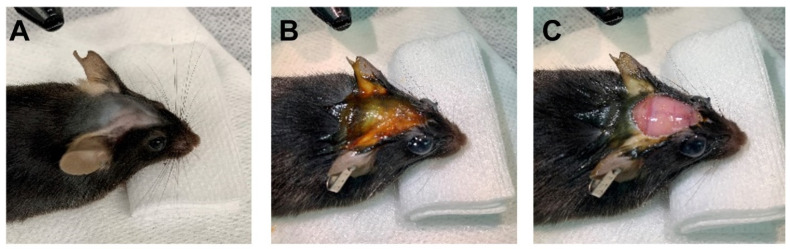
Initial surgery steps. (**A**). After placing the mouse on the heatpad and ensuring appropriate anesthetic depth, shave the hair on the head. (**B**). Clean with betadine and alcohol prior to cutting and apply eye ointment. (**C**). Cut and remove the scalp.

**Figure 3:**
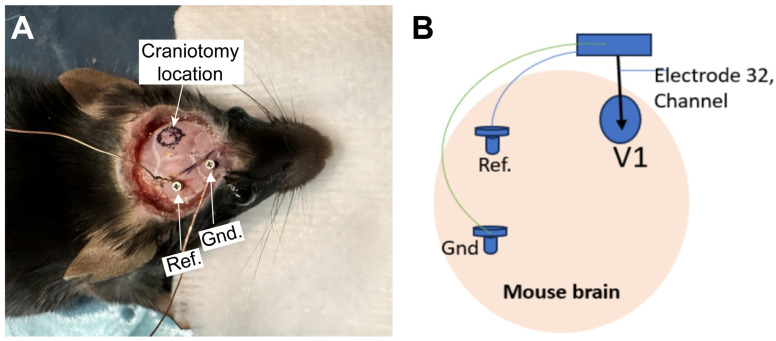
(**A**). After removing periosteum and muscle, seal the wound margins with Vetbond and mark the craniotomy location. Then connect the ground (Gnd.) and reference (Ref.) screws and wires to the contralateral side of the craniotomy. (**B**). Schematic of the connection of the Gnd. and Ref. wires with the probe.

11. Induce anesthesia. Administer the anesthetic solution via intraperitoneal (IP) injection (0.12 mL/30 g weight) and wait ~5 min. Anesthetic depth can be assessed by tail pinch.
12. Administer Buprenorphine subcutaneously from the back, near the neck (1 mg/kg).
13. Shave the head from the eyes to the neck area (Figure 2A), clean it with Betadine solution and apply ocular ointment to the eyes (Figure 2B). Using a scalpel blade, make an incision on the midline and remove the scalp (Figure 2C). Remove the muscle from the skull, dry any bleeding, and scrape away the periosteum on the skull surface with a scalpel blade. Seal the surrounding muscles and skin with Vetbond (Figure 3A). This provides additional substrate for the cement to grip, as well as preventing further bleeding.

14.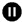 **PAUSE STEP:** Let it dry for 2 min. Ensure that there is no bleeding in the wound margins before applying any cement.
15. **OPTIONAL STEP:** The micro burr can be used to score the skull surface to increase the cement strength.
16. Mark the outline of the craniotomy—around 3 mm diameter. We place our windows over the primary visual cortex (Figure 3A).

### 3.3. Craniotomy and Installing Electrophysiological Equipment and Headplate (30–45 Min)

**Figure 4:**
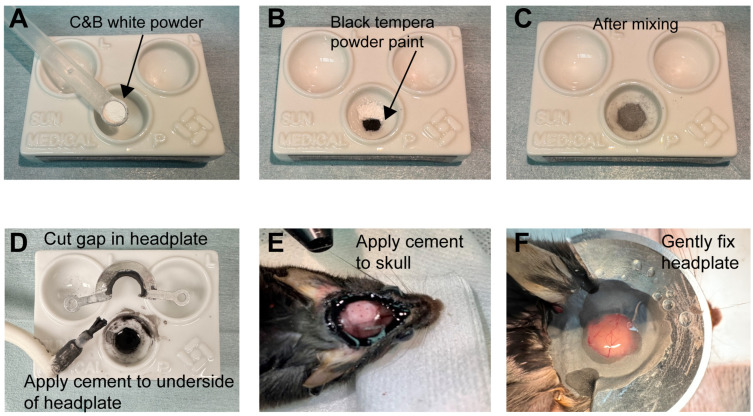
The steps for fixing the headplate. (**A**–**C**): Mixing the cement powder. (**D**–**F**): Applying the cement and fixing the headplate.

**Figure 5:**
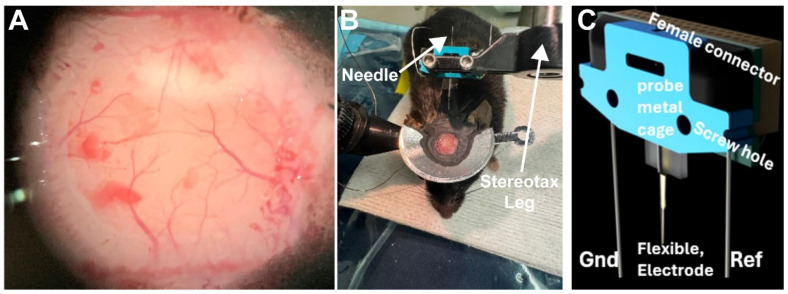
(**A**). The cranial opening showing the exposed tissue of V1 immediately after removing the skull flap. (**B**). Electrode probe mounted on the stereotaxic support. (**C**). Electrode mount diagram.

**Figure 6:**
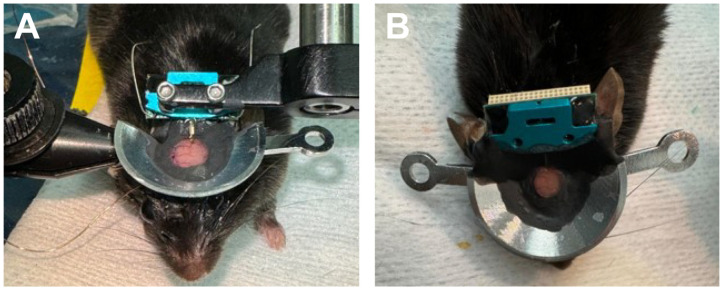
(**A**). An electrode implanted in the V1 region of an example mouse. (**B**). Completed surgery/implantation after connecting the reference and ground wires and filling the gap between the electrode mount and the headplate with cement.

1. Drill two small holes on the contralateral side for the ground (Gnd.) and reference (Ref.) connections (Figure 3A).
2. Cut two small pieces of copper wire and attach one to each screw. Tighten screws into skull.
3. Mix 50% C&B powder and 50% tempera powder paint (Figure 4A–C).
4. After uniformly mixing the C&B powder with the tempera powder paint, add 4 drops of Quick Base and 1 drop of Catalyst, mixing it to the consistency of honey.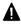 **CRITICAL STEP:** After adding the Catalyst, the cement will cure within a minute and so must be used quickly.
6. Quickly apply it to the bottom of the headplate and around the skull, except the region marked for the craniotomy (Figure 4D,E).
7. Place the headplate on the skull, centered over the craniotomy location, and apply additional cement solution to the inside of the headplate surrounding the marked region (Figure 4F).
8. Hold the headplate in position gently for a few minutes until the Metabond has dried.
9. Drill a circle of bone for the cranial window and gently lift the skull flap with forceps.
10. Apply physiological saline to the exposed region to keep the brain tissue moist and to rinse away any bleeding (Figure 5A). Ensure that no blood sticks to the tissue surface for optimal optical clarity.

11. **OPTIONAL STEP:** The dura can be removed if desired. It is especially important to keep blood from sticking to the pial surface, as it is harder to rinse away than when on the dura.
12. Mount the 32-channel electrode probe (Figure 5C) to the stereotaxic arm support and bend the Gnd and Ref. wires upward so that they will not touch the headplate (Figure 5B).
13.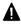 **CRITICAL STEP:** Make sure that the electrode does not get moist before entering or the polymer bond will disintegrate. Holding the electrode over the cortex for even 10 s can be sufficient to weaken the polymer. Do not touch the cortex until entering very rapidly.

14.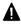 **CRITICAL STEP:** Avoid puncturing large vessels when driving in the electrode.
15. After positioning the electrode guide wire near the entry position, move the stereotax with a speed of 1 mm per second to rapidly insert the electrode affixed to the metal needle into the brain to avoid disassociation of the electrode from the guide needle (see Results).
16.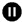 **PAUSE STEP:** Wait 4–5 min for the polymer holding the electrode to the guide needle to dissolve, allowing the electrode to separate.
17. Remove the metal guide needle.
18. Aspirate and dry any remaining saline. Place the cover glass gently over the exposed cortical tissue with the beveled gap positioned above where the electrode leaves the cranial opening.
19. Seal the cover glass with Loctite adhesive (5E208).
20. Apply Kwik-Sil silicon to fill in the space between the electrode mount and the headplate.
21. Solder the ends of the copper wires from the screws to the Gnd. and Ref. wires coming off the electrode probe (see Figure 3B for schematic of connections).
22.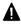 **CRITICAL STEP:** After soldering the wires, check the continuity of connections, using the digital multimeter. Any open connection will result in the loss of electrophysiological signals in the experiment.
23. Prepare a new batch of cement, as in steps 6–7, and apply around the region to fully secure the electrode mount, filling any space between the mount and the headplate (Figure 6A,B).
24. Wait 5–10 min for the cement to dry.
25. Administer the anesthetic antidote (Antisedan, IP) before putting the mouse in the cage.
26. Allow the animals to recover before imaging vascular and neuronal activity. We typically wait one week to ensure that the animals have recovered their strength to endure another manipulation and that there are no signs of infection or anything that could impede the success of the experiments.
27. More information about the electrode is provided in Supplementary Materials S1, Link1.

### 3.4. Setting up (20–30 Min) and Running the Experiment

**Figure 7:**
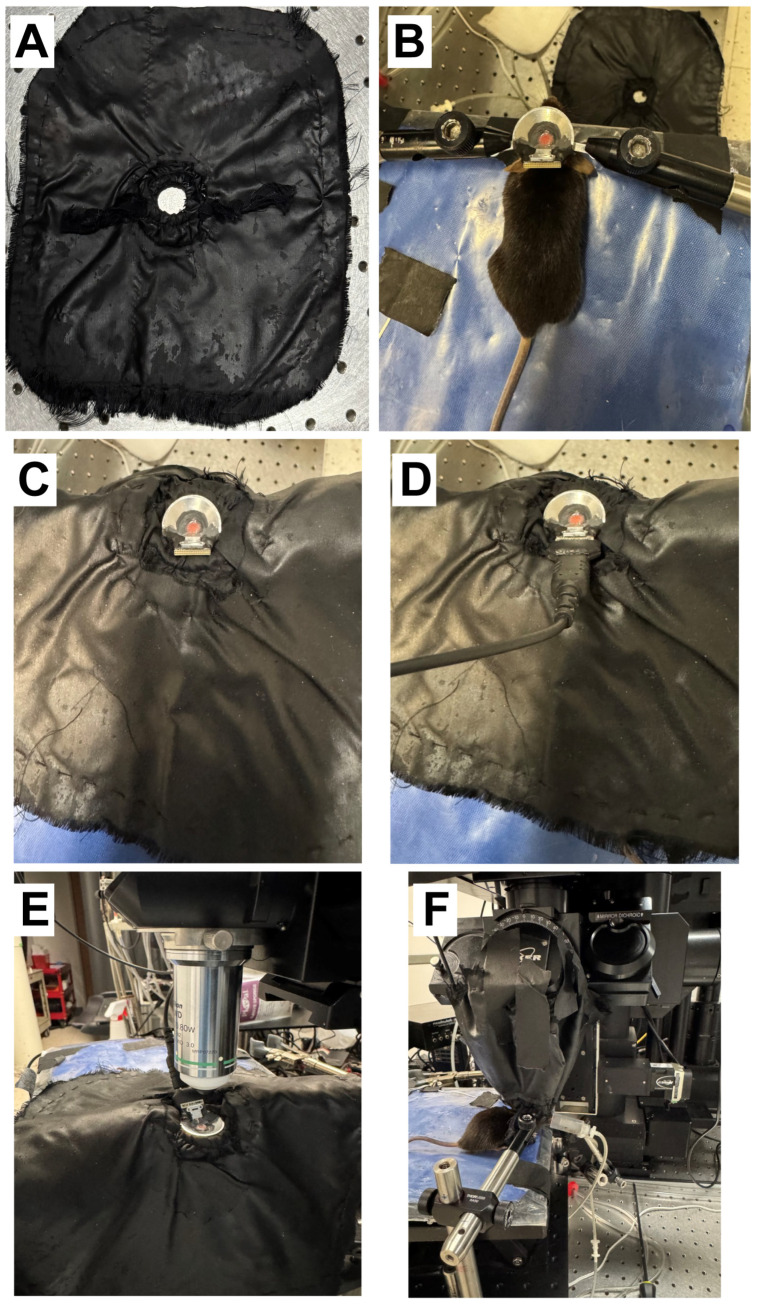
The steps for setting up the combined imaging/electrophysiology/optogenetics experiment. (**A**). The double-layered cloth with cutout for securing around headplate. An elastic band is sewn into the cloth around the hole, which can be cinched to make a tight light seal below the headplate. (**B**). The mouse with headplate fixed to holder bars. (**C**). The black cloth is positioned over the bowl-shaped headplate. (**D**). The electrode wiring is connected. (**E**). The animal is positioned under the objective. (**F**). The cloth is wrapped up and over the objective while remaining below the headplate for optimal shielding.

The custom-designed headplate provides support to fix the head of the mouse during imaging sessions, as well as serving as a reservoir for the water for the objective. The two legs of the headplate can be tightened to the arms of the supporting clamps attached to the animal stage (Figure 7B). Covering the outer portion of the headplate and the objective with black-out cloth provides shielding from environmental light interference during imaging (Figure 7C–F).

1. After the mouse has sufficiently recovered, bring the animal to the imaging room, place on the heat pad, and anesthetize. We use isoflurane in air (≤0.8%) and ~30 µL chlorprothixene (1 mg/mL, intramuscular).
2. Insert the headplate between the adjustable supporting arms and tighten (Figure 7B).
3. We use a custom-designed cloth made by sewing two layers of blackout cloth together with a hole in the center of each layer. A band of elastic is sewn between the two layers that are adjacent to the hole with the two ends of the band coming through the bottom layer of cloth (Figure 7A). This fits over the headplate and the elastic bands are then pulled to tighten the hole around the headplate, leading to excellent light shielding (Figure 7C).
4. Connect the electrode probe to the head stage (UH32) male connector for electrophysiology (Figure 7D).
5. Place the objective lens for two-photon imaging (Figure 7E).
6. Wrap the custom-designed cloth around the electrode and objective lens and secure it with black tape. Continue using black cloth and tape as necessary to ensure that no space is left for environmental light to pass through and interfere with the emitted fluorescence light (Figure 7F).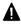 **CRITICAL STEP:** Check for any openings around the covering through which environmental light may enter and use tape to cover the openings. Switch off the imaging room lights.
8. The UH32 is connected to the MCU, which is in turn connected to the ECU and the computer. Connection diagrams are provided in Supplementary Materials S1 Links 2 and 3.
9. After all the cables are connected, turn on the MCU and ECU. The MCU has an LED, which will be green if it detects a connection with the headstage (orange if not).
10. Open the Trodes 2.5.3 software on the computer connected to the MCU to collect the electrophysiology data. See Supplementary Materials S3 for a flowchart.
11. Run the Prairie View software on a second computer dedicated to collecting two-photon imaging data. Steps to collect data using Prairie View are provided in Supplementary Materials S1 Link 4.
12. BNC cables can be used to connect the ECU to either computer to control the experiment and monitor the environment as desired.
13. Under deep anesthesia (2.5% isoflurane), fluorescent dextrans can be injected infraorbitally to visualize the vasculature during imaging.
14. We presented visual stimuli, using the Psychophysics toolbox extensions [33]. The visual stimuli consisted of square-wave gratings, presented at 100% contrast, 2 Hz, and 0.05 cycles/degree, as described previously [19,32].
15. Optogenetic stimulation was provided by a 590 nm LED. Additionally, 100 ms pulses were presented at around 2.5 Hz. The power was controlled using an LED driver to deliver ~18 mW/cm^2^, as measured at the objective. Our previous work showed that this constricts virtually all the vessels in the field of view [25]. Supplementary Figure S2 shows the arrangement of the components of the light path, including light sources, filters, and detectors.

### 3.5. Software Used in This Protocol

A detailed list of all software used in this protocol is listed in Table 5. We tried to use publicly available open-source software as much as possible so that the protocol can be replicated by researchers around the world. Electrophysiology data recording and analysis are performed using all open-source software. Two-photon data were recorded using ‘Prairie View’ software 5.8, provided by the Bruker ^®^ company. 

### 3.6. Data Analysis

#### 3.6.1. For Electrophysiology Data

1. Before recording the raw data using Trodes, follow the steps in Supplementary Materials S1 Link 2, or open the Trodes software in a browser.
2. During the recording, enter the parameter values of the low-pass and high-pass filters. We use 0–200 Hz for the low-pass (LFP data) and 300–6000 Hz for the high-pass (spiking data) filter.
3. Low-pass filtered data is analyzed with Fourier transform functions (see Results).
4. The high-pass filtered data (for spiking) is exported as a DAT file to Kilosort.
5. Within the Kilosort software, input the electrode configuration and experimental parameters, e.g., number of channels = 32, probe type = tetrode or linear, and sampling rate = 30 kHz. We used the default values for the advanced settings, but these can be adjusted if desired.
6. After completion of the Kilosort processing, spike templates, times, cluster IDs, and other parameters will be saved for processing in Phy, in files such as params.py.
7. Within the Phy software, read the params.py file and modify the spike sorting as desired. We visually inspected the spike shape, looked for dips at zero in the inter-spike-interval histogram, and filtered based on amplitude, e.g., if the amplitude was below <10 micro volt, we labelled that spike template as noise.
8. Use MATLAB or Python to plot the results. Note that both Kilosort and the Phy software are built in Python.

#### 3.6.2. For Two-Photon Imaging Data

1. During recording, set the imaging acquisition parameters. We typically image in Resonant mode, averaging 8 frames. We typically record at the surface and in layer 2/3 by rapidly changing the focal plane with an electro-tunable lens (ETL). This leads to a frame period at each plane of around 0.776 s.
2. The PrairieView software records the data as a set of ome.tiff images. These can be imported into software like Matlab/Python for further analysis.
3. We load images into the MATLAB environment and process the frame-by-frame diameter of different vessels in the field of view using the brightness profile across hand-drawn cross sections of the vessels. This has been described in detail with the sample code provided in earlier publications [25].

## 4. Results

**Figure 8:**
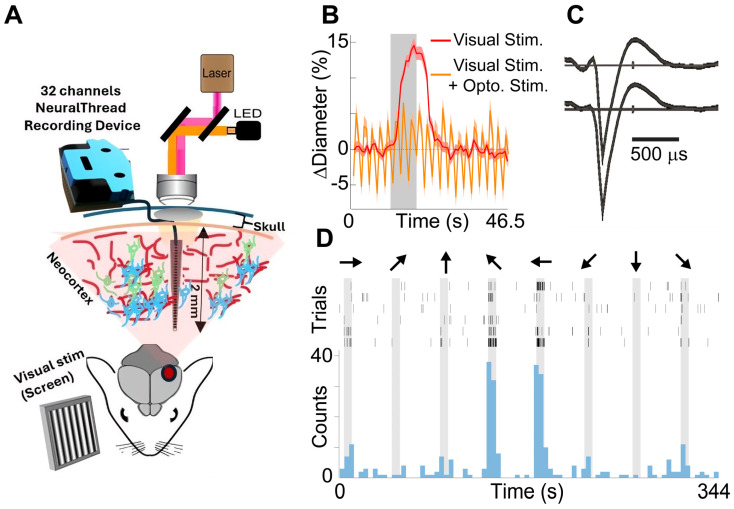
(**A**). Schematic of the method. (**B**). Stimulus-evoked increase in the diameter of blood vessels (red) was eliminated with concurrent optogenetic stimulation (orange). Data are from two consecutive runs in the same animal. The averages of 6 trials of 8 stimulus orientations are plotted. Gray band indicates visual stimulus interval. Optogenetic stimulation was a 100 ms flash from a 594 nm LED every 2.3 s. This led to oscillations in the diameter of the vessels as they would begin to relax back to baseline before being constricted again. (**C**). Neuron spike shape detected on two adjacent channels of the electrode. (**D**). Single neuron activity response to 8 orientations of visual stimulus (drifting gratings). **Top**: Raster plots of 6 repetitions of the stimulus sequence. **Bottom**: Spike count histogram. Gray bands are stimulus intervals. Arrows indicate the direction of the drifting grating stimuli.

**Figure 9:**
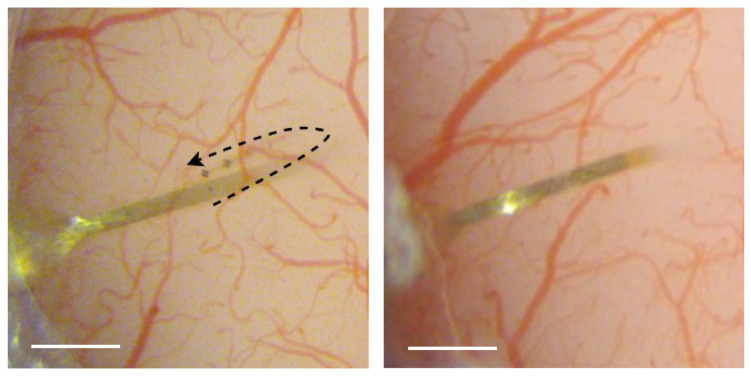
Electrode implantation without removing the dura mater (**left**). The electrode is seen as the golden ribbon; the dotted line illustrates the folding trajectory of the electrode after a faulty implantation. In a second mouse, (**right**) the dura was removed and the electrode implanted cleanly.

We combined electrophysiology and two-photon imaging to simultaneously measure neuronal and vascular activity (Figure 8A). The presentation of drifting grating visual stimuli reliably produced vasodilation (Figure 8B), and in many of the single units we recorded (Figure 8C), increases in action in the potential firing above the baseline (Figure 8D). In a subset of experiments, optogenetic stimulation of blood vessels was also incorporated (Figure 8B; [25]). We implanted electrodes in thirteen mice with the dura left intact. In five of the animals, there was dissociation of the guide pin from the electrode during the insertion, leading to bending or incomplete insertion (Figure 9). In contrast, in three mice we removed the dura prior to implantation and all electrodes were successfully inserted without folding or bending. We were concerned that removal of the dura may hasten the degradation of the imaging window. One of the three mice died prior to imaging, but the other two still have excellent optical clarity at 3 and 5 months, respectively. In comparison, in the mice with the intact dura, optical clarity was good up until at least 4 months in eight of the 9 mice imaged to that duration. Three of these mice had useable windows up to or just beyond 5 months, and one lasted around one year before losing clarity.

**Figure 10:**
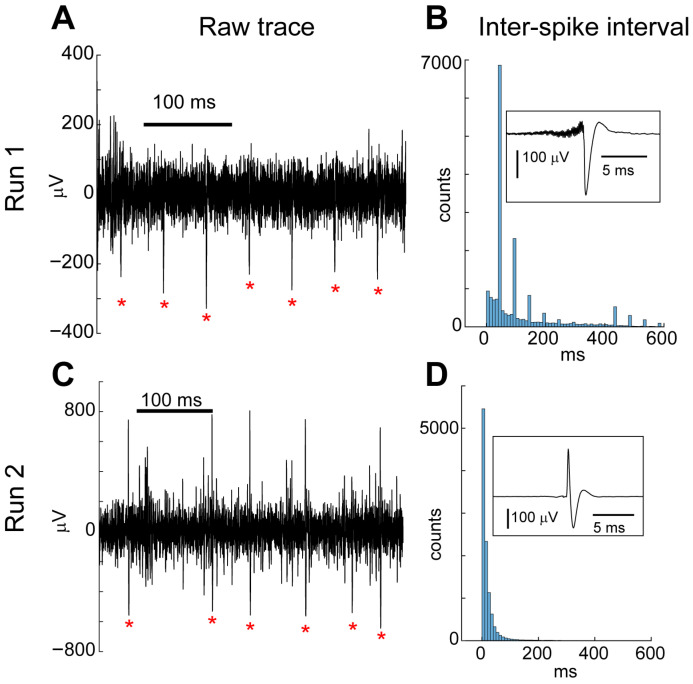
(**A**). Two-photon laser light hitting the electrode causes an artifactual waveform at regular intervals. A 400 ms window of the raw voltage trace of an example run is shown, with red asterisks indicating detected spikes matching the template in (**B**). (**B**). The inter-spike-interval distribution had a large peak ~50 ms, matching the imaging frame rate (~21 Hz). Peaks at harmonics of 50 ms indicate when one or more laser artifact “spikes” were missed. The inset shows the average waveform with an unnatural ringing in the trace, likely caused by the high frequency resonant mirror used for imaging. (**C**). A 400 ms window of raw voltage from a subsequent run when the electrode was positioned just outside the imaging window. Detected spikes (red asterisks) occurred at irregular intervals. (**D**). The inter-spike-interval showed a standard Poisson-like distribution and the average waveform had no irregular features.

**Figure 11:**
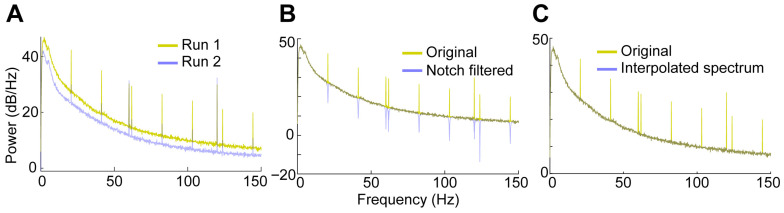
(**A**). The power spectrum of the LFP data also showed the effects of the laser artifact at regular intervals. The ~21 Hz imaging rate and its harmonics, in addition to the standard 60 Hz electrical interference artifact, showed peaks of increased power. In run 2, the 21 Hz artifact was greatly reduced. (**B**). Notch filtering at the key frequencies reduced the power, leaving a trough in the spectrum. (**C**). For averaging within frequency bands, linear interpolation across the peaks is an effective tool for artifact removal. The duration of the runs being analyzed was 2442 s for run 1 and 2395 s for run 2.

Because this preparation combines two-photon imaging, optogenetics, and electrophysiology, we checked for the presence of light-induced electrical artifacts [34]. We observed that when the two-photon infra-red laser hit the electrode, a high-pitch sound was heard through the speaker connected to the electrical output. This noise created a distorted waveform that was extracted by the automated spike detection algorithm as a putative action potential (Figure 10A,B). The events matching this template exhibited a highly regular inter-spike interval of approximately 50 ms, corresponding to the ~21 Hz imaging frame rate (Figure 10A,B). In contrast, true action potentials showed undistorted waveforms and had typical Poisson-like distributions of inter-spike intervals (Figure 10C,D). The laser artifact was also reflected in the local field potential (LFP), appearing as an increase in power at 21 Hz and its harmonic frequencies (Figure 11A, yellow trace). When the electrode was positioned just outside the imaging window, no artifactual spikes were detected and the LFP artifact was greatly reduced (Figure 11A, blue trace). To remove the artifact from the LFP data, we built notch filters using the frequency peaks that were automatically detected from the power spectrum. Applying the notch filtering to the raw LFP signal eliminated these artificial peaks (as well as the standard 60 Hz electrical noise), leaving a drop in the power spectrum at those frequencies (Figure 11B).

**Figure 12:**
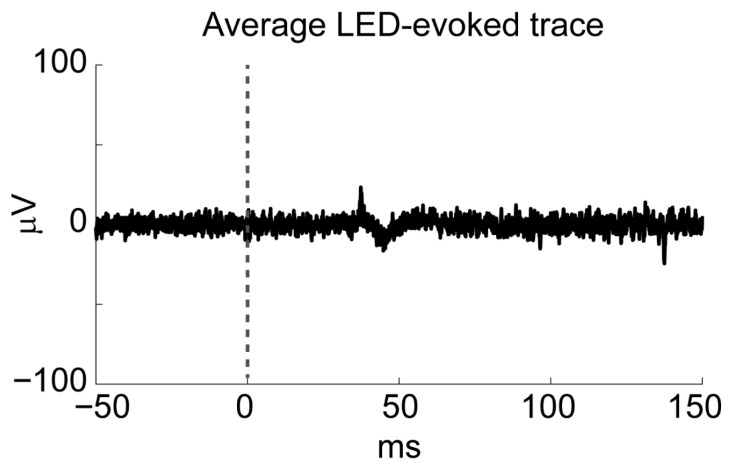
Average of a 200 ms window of raw voltage traces aligned to the LED onset command pulse (average of 702 LED flashes). The LED flashed for 100 ms.

We next determined whether the LED used for optogenetic stimulation produced any electrical artifact. No noise was detected during the recording matching the LED frequency. Aligning and averaging the raw electrical trace to the LED onsets revealed only a minimal artifactual signal which never resembled an action potential waveform and was not detected by the spike-sorting algorithm (Figure 12). Thus, the LED does not appear to introduce artifacts that could be mistaken for neuronal spiking activity. Note that the LED artifact seems delayed by around 40 s from the time that the command pulse is sent (time zero). This is due to the delay in the system to allow for a mechanical shutter to close in front of the highly sensitive photomultiplier tube (used for two-photon imaging light collection). The LED is so bright that even if a very small percentage of the light came through to the detectors, they could quickly be damaged.

## 5. Discussion

We have provided a step-by-step experimental protocol for combining electrophysiology with two-photon optical imaging and optogenetics. This experimental preparation is readily compatible with other methods for monitoring blood flow, including laser speckle contrast imaging and intrinsic signal imaging. Additionally, the imaging of cellular activity could be included along with the electrical recordings, either at two-photon or mesoscale resolutions. Thus, this approach provides a powerful means to combine electrical recordings with a wide range of imaging methods. In addition, while we have only used this procedure in mice, it should be applicable in other animal models as well.

Removal of the dura prior to electrode implantation led to an improved success rate. However, for chronic imaging windows, the removal of the dura presents potential drawbacks, including inflammation and re-growth of tissue which can make the windows cloudy and opaque. Moreover, certain studies may require an intact dura for the experimental question, e.g., studies of CSF influx. For experiments in which the dura must remain intact, several factors are likely critical to successful implantation. First, exposure of the electrode to moisture must be avoided. Slowly positioning the electrode over the cortex can lead to moisture absorption, weakening the polymer that holds the electrode to the guide pin. Similarly, contact with the CSF prior to the high-speed insertion will also increase the likelihood of the electrode dissociating from the guide pin and folding upon entry. In our configuration, the electrodes were inserted at an angle, with the electrode below (closer to the cortex than) the guide pin. Inverting this orientation, so that the guide pin punctures the dura immediately before electrode insertion, may help to prevent dissociation. Nonetheless, removing the dura eliminates a major source of mechanical resistance, and so, when possible, it is recommended to maximize implantation success.

The scanning laser introduced prominent artifacts. We have found that the best approach for dealing with artificially produced spikes is to have robust screening to detect them and then remove them from the dataset. It is possible that they could be filtered out of the signal by, for instance, template matching and subtracting. But this risks distorting the signal and affecting other true action potentials. The unique spike waveform and the telltale inter-spike-interval distribution (Figure 10A,B) allow for a clear identification of the laser-induced artifacts.

For the LFP data, the spikes in power at the imaging frequency and its harmonics are very sharp and clear. We applied notch filtering, as is often done for 60 Hz electrical noise, and this created drops in the power spectrum at these frequencies. However, this has little advantage over the original power spectrum, as there are still clear artifacts (dips instead of peaks). For comparing across runs or conditions, these very sharp peaks can simply be ignored. However, a common practice in LFP analysis is to pool frequencies into different bands (delta, gamma, etc.) and compare across conditions. To avoid contamination from these sharp peaks in this type of analysis, we simply linearly interpolate across the peaks in the power spectrum itself (Figure 11C).

One limitation of the electrodes that we use is that they are more susceptible to photovoltaic artifacts than other designs. Several studies have developed electrodes with special properties to reduce the impact of light artifacts [26,27,28,35]. However, to our knowledge, these products all contain only surface contacts, preventing single-unit isolation in deep cortical layers. The nanelectronic thread electrodes we use here trade greater susceptibility to light artifacts for the ability to be driven into the cortex with minimum tissue trauma and prolonged stable recording. To avoid contamination from light artifacts, our recommendation is to avoid scanning directly over the electrode when possible, and to use robust screening to identify and remove the artifactual signals when they do appear.

## Figures and Tables

**Table 1:** Surgery materials.

Critical Component Name	Provider Name	Catalog Number
Surgical Microscope	Zeiss (Oberkochen, Germany)	Stemi 2000
Heat-pad and PhysioSuite	Kent Scientific (Torrington CT, USA)	PS-03
Student Fine Scissors	Fine Science Tools (Foster City CA, USA)	91460-11
Syringe u100 ½ cc	Fisher (Hampton NH, USA)	1482679
Fine Scissor-Tungsten	Fine Science Tools (Foster City CA, USA)	14558-09
Student Scalpel Handle—#3	Fine Science Tools (Foster City CA, USA)	91003-12
Scalpel Blade #15	Fisher Hampton NH, USA	22079693
Student Dumont #5CO Forceps	Fine Science Tools (Foster City CA, USA)	11295-20
Dumont #3 Forceps	Fine Science Tools (Foster City CA, USA)	11293-00
Measuring Ruler	Fisher (Hampton NH, USA)	S40641
Soldering Station	Weller (Pleasant Prairie WI, USA)	we-1010, 120 v
Headplate	Custom designed in the Lab	Not applicable
Drill Burr 0.05 mm	Fine Science Tools (Foster City CA, USA)	19007-05
Cover Glass 3 mm	Warner instruments (Holliston MA, USA)	64-0720
Cover Glass 4 mm	Warner instruments (Holliston MA, USA)	64-0724
UV-cured Optical Adhesive	Norland (Jamesburg NJ, USA)	NOA 61
Grindstone Drill Bit	Misumi USA Inc. (Schaumberg IL, USA)	CA1063
Saline 0.9% 250 mL	Patterson Vet (Loveland CO, USA)	78696640
Loctite 416 Adhesive Gel	Grainger (Augusta GA, USA)	5E208
Silicon Adhesive	World Precision Instruments (Sarasota FL, USA)	Kwik-Sil
Hot Bead Sterilizer	Fine Science Tools (Foster City CA, USA)	18000-45
Vetbond	Fisher (Hampton NH, USA)	NC9259532

**Table 2:** Liquid compounds and materials.

Critical Component Name	Provider Name	Catalog Number
Buprenorphine	Covetrus (Portland ME, USA)	72117
Ocular Ointment	Patterson Vet (Loveland CO, USA)	78444656
C&B Metabond	Parkell (Edgewood NY, USA)	S380
Dexamethasone	Patterson Vet (Loveland CO, USA)	78944526
Ketamine	Patterson Vet (Loveland CO, USA)	78908598
Dexdomitor	Patterson Vet (Loveland CO, USA)	78677105
Isoflurane	Patterson Vet (Loveland CO, USA)	78932374
Chlorprothixene	Sigma (St. Louis MO, USA)	C1671
Antisedan	Patterson Vet (Loveland CO, USA)	78677097
Betadine solution	Patterson Vet (Loveland CO, USA)	78363379
Saline solution	Patterson Vet (Loveland CO, USA)	78696640
Tempera powder paint	Amazon (Seattle WA, USA)	1738
Stoelting dental cement kit	Fisher (Hampton NH, USA)	10000786
Texas red dextran	Fisher (Hampton NH, USA)	D1830
FITC dextran 2000 kD 100 mg	Sigma (St. Louis MO, USA)	FD2000S

**Table 3:** Electrophysiology recording system.

Critical Component Name	Provider Name	Catalog Number
32-channel flexible electrodes	SpikeGadgets (San Francisco CA, USA)	910-00001-A
32-channel head stage (UH32)	SpikeGadgets (San Francisco CA, USA)	860-00004-A
Head stage signal converter	SpikeGadgets (San Francisco CA, USA)	HCU
General purpose commutator	SpikeGadgets (San Francisco CA, USA)	Comp_2
Main control unit (MCU)	SpikeGadgets (San Francisco CA, USA)	MCU_3
Environmental control unit (ECU)	SpikeGadgets (San Francisco CA, USA)	ECU_2
Micro HDMI cable	SpikeGadgets (San Francisco CA, USA)	None
HDMI cable	SpikeGadgets (San Francisco CA, USA)	None
Omnetics nano extension wire	DigiKey (Thief River Falls MN, USA)	A79029-001
Fluke digital multimeter	Grainger (Augusta GA, USA)	Flucke-15B+
BNC cable	Thorlabs (Newton NJ, USA)	CA3124

**Table 4:** Critical components for the two-photon imaging system.

Critical Component Name	Provider Name	Catalog Number
Optical table with tuned damping	Newport (Irvine CA, USA)	RS 2000
Two-photon pulsed laser	Spectra-Physics (Milpitas CA, USA)	Insight X3
Two-photon imaging system	Bruker (Billerica MA, USA)	Ultima 2P plus
Nalco 460 PCCL 104 coolant	Spectra-Physics (Milpitas CA, USA)	1607-0546
Plate Clamp Holder	ThorLabs (Newton NJ, USA)	PC2
Black tape	ThorLabs (Newton NJ, USA)	T743-2.0
Blackout cloth	ThorLabs (Newton NJ, USA)	BK5
Light-emitting diode (LED)	ThorLabs (Newton NJ, USA)	M590L4
LED driver	Thorlabs (Newton NJ, USA)	LEDD1B

**Table 5:** List of software used.

Software Name	Use	Availability
Tinker CAD^®^	Design 3D object	Open source
Trodes 2.5.3 Spikegadgets^®^	Electrophysiology recording	Open source
Kilosort^®^ 2	Neuron spike sorting	Open source
Phy 2^®^	Neuron spike curation	Open source
Prairie View^®^ 5.8	Two-photon data recording	Bruker
Matlab 2024B^®^	Post data processing	Matlab
Python 3^®^	Post data processing and visualization	Python

## Data Availability

The raw data included in the results of this article will be made available by the authors upon request.

## Supplementary Materials

The following supporting information can be downloaded at: https://www.mdpi.com/article/10.3390/mps9030068/s1. Supplementary Material S1: Links to webpages for electrophysiological and imaging equipment information; Supplementary Material S2: Headplate CAD file; and Supplementary Material S3: Electrophysiology data collection flow chart. Supplementary Figures: Supplementary Figure S1: Diagram of the headplate; Supplementary Figure S2: Diagram of the light path. PMT (photomultiplier tube); BP (band-pass filter); Supplementary Figure S3: Gantt chart of the timing of each section.

## Abbreviations

The following abbreviations are used in this manuscript:

LFP	Local field potential
UH32	32-channel head stage
MCU	Main control unit
