# Supplementary material for: Simultaneous In Vivo Electrophysiology, Two-Photon Imaging, and Optogenetics for Probing Neurovascular Coupling
Source: Methods Protoc. 2026 Apr 25;9(3):68. doi: 10.3390/mps9030068 (PMC13214910; doi:10.3390/mps9030068)

Supplementary Figures

Supplementary Figure S1: Diagram of the headplate.

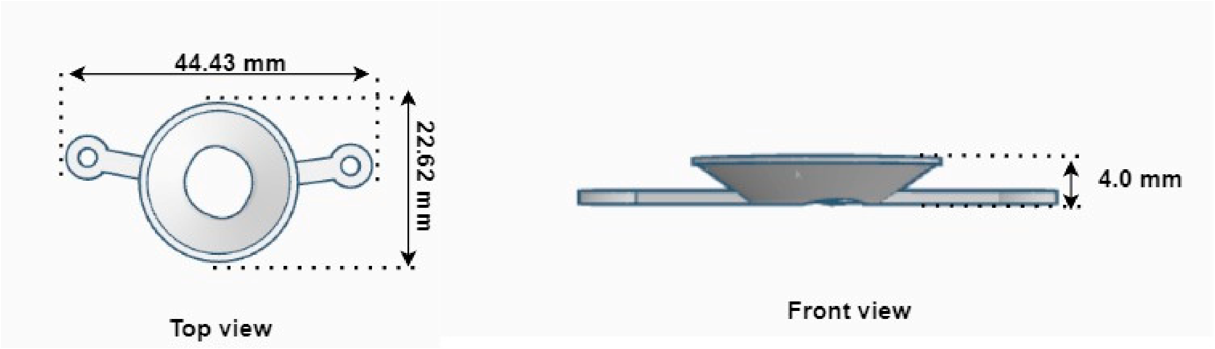

Supplementary Figure S2: Diagram of the light path. PMT (photomultiplier tube); BP (band-pass filter)

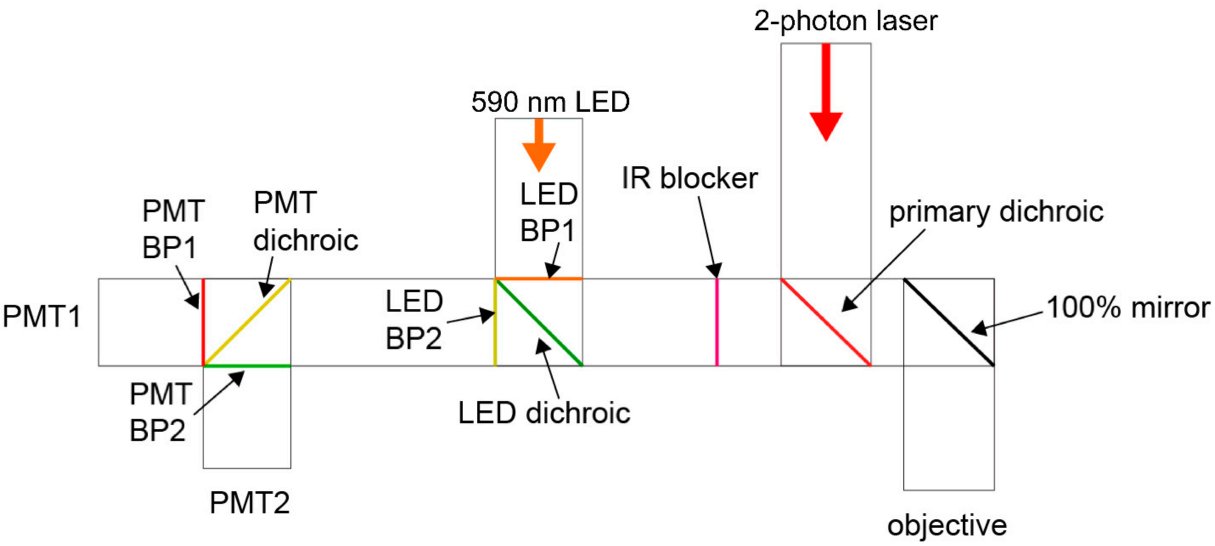

Supplementary Figure S3: Gantt chart of the timing of each section

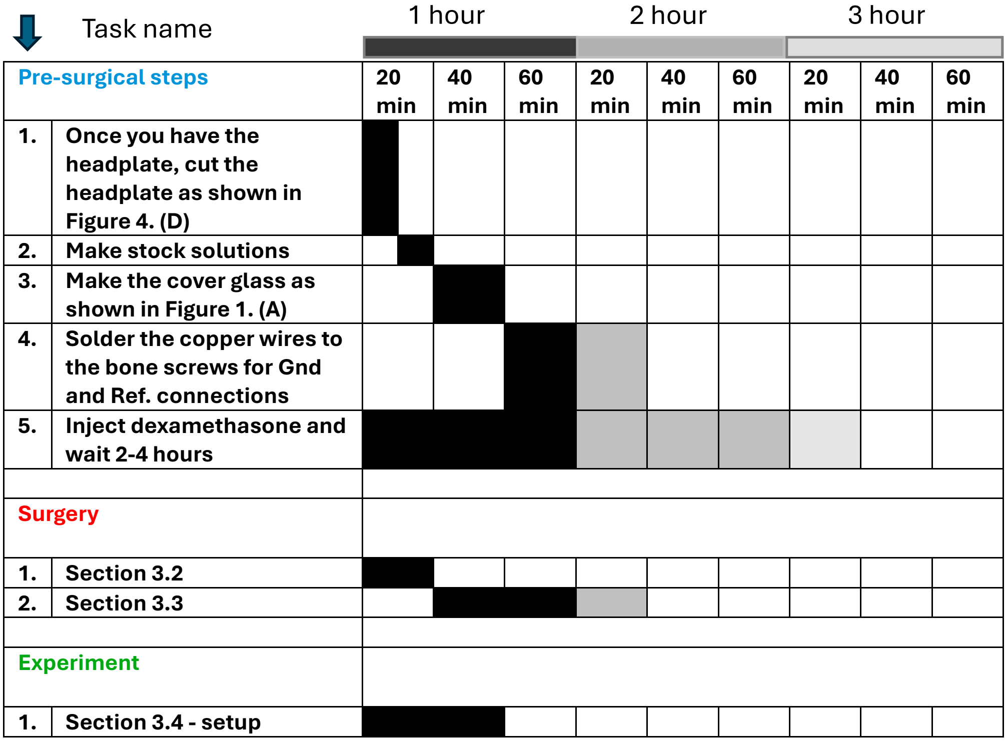

Supplement: Supplementary file 1 [file mps-09-00068-s001.zip › mps-4194374-supplementary-Figure.pdf]
