# Supplementary material for: Simultaneous In Vivo Electrophysiology, Two-Photon Imaging, and Optogenetics for Probing Neurovascular Coupling
Source: Methods Protoc. 2026 Apr 25;9(3):68. doi: 10.3390/mps9030068 (PMC13214910; doi:10.3390/mps9030068)
Supplement: Supplementary file 1 [file mps-09-00068-s001.zip › Supplementry-Materials-S1.pdf]

Supplementary materials S1

Link 1:

https://neuralthread.com/

Link 2: Documentation for the electrophysiology recording software “Trodes”.

https://spikegadgets.com/documentation/

Link 3: Electrophysiology hardware connection schematics

https://spikegadgets.wpenginepowered.com/wp-content/uploads/2021/06/setup-guide.pdf

Link 4: Two-photon microscope Operator’s Manual

https://cudmore.github.io/images/Ultima-Operators-Manual-Rev-B.pdf
