# Supplementary figures and images for: Simultaneous In Vivo Electrophysiology, Two-Photon Imaging, and Optogenetics for Probing Neurovascular Coupling
Source: Methods Protoc. 2026 Apr 25;9(3):68. doi: 10.3390/mps9030068 (PMC13214910; doi:10.3390/mps9030068)

# Steps during collecting data using Trodes

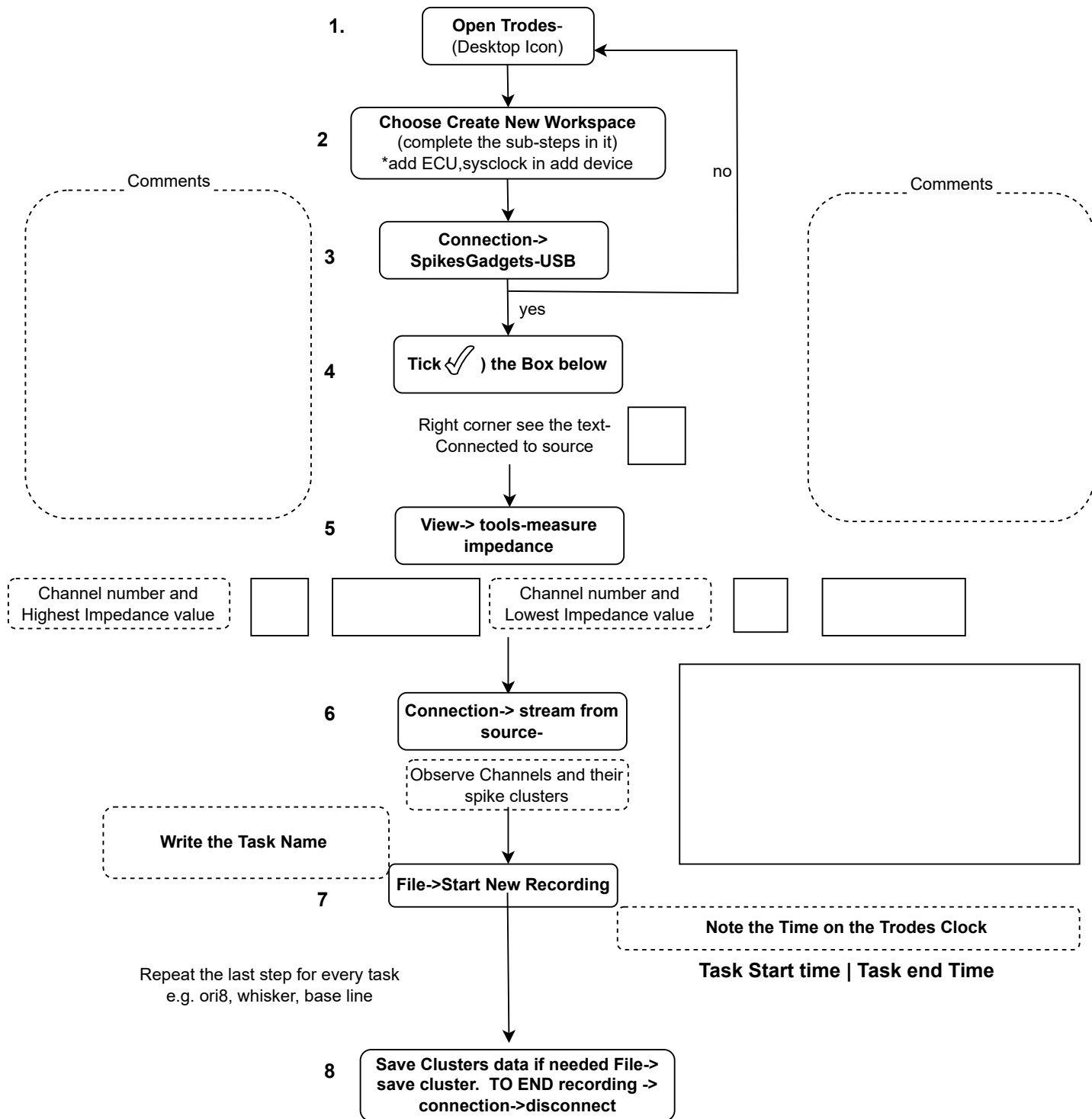

Supplement: Supplementary file 1 [file mps-09-00068-s001.zip › S3-(Electrophysiology data collection flow chart).pdf]
